# Patient preference of level I, II and III sleep diagnostic tests to diagnose obstructive sleep apnoea among pregnant women in early to mid-gestation

**DOI:** 10.1007/s11325-024-03114-0

**Published:** 2024-08-21

**Authors:** Frances Clements, Hima Vedam, Yewon Chung, Nathaniel S. Marshall, Kerri Melehan, Annemarie Hennessy, Angela Makris

**Affiliations:** 1 Department of Respiratory and Sleep Medicine, Liverpool Hospital, South Western Sydney Local Health District, Locked Bag 7103, Liverpool BC, NSW 1871 Australia; 2 School of Medicine, Western Sydney University, New South Wales, Australia; 3 Women’s Health Initiative Translational Unit, Ingham Institute for Medical Research, South Western Sydney Local Health District, New South Wales, Australia; 4 School of Clinical Medicine, Discipline of Medicine, South Western Sydney Clinical Campuses, UNSW Sydney, New South Wales, Australia; 5 Woolcock Institute of Medical Research, Centre for Sleep and Chronobiology, Macquarie University, Sydney, NSW Australia; 6 Department of Health Sciences, Macquarie University, Sydney, Australia; 7 Faculty of Medicine and Health, University of Sydney, Sydney, Australia; 8 Department of Respiratory and Sleep Medicine, Royal Prince Alfred Hospital, New South Wales, Australia

**Keywords:** Preeclampsia, Gestational diabetes mellitus, Level I, Level II, Level III

## Abstract

**Purpose:**

There is a paucity of data on preferences for obstructive sleep apnoea (OSA) diagnostic tests during pregnancy. Simple test completion rates fail to capture patient preference or experience of completing sleep diagnostic tests. We assessed preference, ease of use, convenience, and the repeatability of level I, II and III sleep diagnostic tests, using questionnaires, in pregnant women in early to mid-gestation.

**Methods:**

Pregnant women with signs or symptoms of OSA, or at high risk of cardiometabolic disorders of pregnancy completed level I, II and III sleep studies by 24 weeks gestation. Participants then completed a questionnaire to rank test preference. Additional questionnaires assessed ease of use, convenience, and acceptability to repeat test, using 5-point Likert scale questions, yes/no response and optional linked text fields.

**Results:**

Of fifty-two consented participants, 43 completed any questionnaire (mean age 32.7 ± 5.4 years, BMI 32.7 ± 5.4, median gestation at Level I polysomnography (PSG) of 14.2 weeks (interquartile range (IQR) 13.5–17.6)). Of the 29 respondents who completed test ranking questionnaire, level III was the preferred test ((*n* = 21 / 29, 75%)), followed by level 1 (*n* = 6 / 29, 20.7%) and level II (*n* = 2 / 29, 7.1%) (p for diff < 0.001). Level III was reported the easiest test (very easy to complete) (*n* = 16, 51.6%), followed by level I(*n* = 10, 33.3%), and level II (*n* = 9, 9.1%) (p for diff < 0.001)). Level III was reported most convenient test (very convenient to complete) (*n* = 16, 51.6%), followed by level I (*n* = 4, 13.3%) and level II (*n* = 4, 13.3%) (p for diff < 0.001)). Level III was reported most acceptable to repeat (very acceptable to repeat) (*n* = 13, 41.9%), followed by level I (*n* = 3, 10.0%) and level II (*n* = 3, 10.0%) (p for diff < 0.001)).

**Conclusion:**

Pregnant women being assessed for OSA by 24 weeks gestation preferred to undertake level III sleep studies and found level III easier to use, more convenient and most acceptable to repeat than Level I and II studies. Given autonomy is an important principle, patient preference of sleep diagnostic tests should be taken into consideration in sleep clinical services and research involving pregnant women.

**Supplementary Information:**

The online version contains supplementary material available at 10.1007/s11325-024-03114-0.

## Introduction

### Background rationale

There is a growing interest in the diagnosis of obstructive sleep apnoea (OSA) in pregnancy due to the association between OSA and complications in pregnancy such as preeclampsia and gestational diabetes mellitus (GDM) [1]. A recent consensus guideline has recommended the screening of pregnant women for OSA in those with either a body mass index (BMI) 30 or greater, history of hypertensive disorders of pregnancy or gestational diabetes in the current or a previous pregnancy [2]. This screening is recommended in the early to mid-gestation period (gestational weeks 6–28). This allows for the opportunity to initiate OSA treatment in those with the condition, and adequate time prior to surgical intervention in those at risk of anaesthetic complications. The burden associated with undertaking a diagnostic test may be of particular relevance in pregnant women, as pregnancy itself can create a burden on the women, particularly in obese patients [3], in whom the risk of OSA is elevated [4] and therefore the need for testing is increased. Therefore, considering a pregnant woman’s preferences for sleep diagnostic test type may reduce test burden on those at risk of OSA who require testing.

Traditional questionnaire-based screening tools such as STOP BANG and the Epworth sleepiness scale (ESS) have a notably poor performance in detecting sleep apnoea in pregnancy [5–8]. A screening questionnaire to predict risk of OSA in pregnant women based on a history of chronic hypertension, pre-gestational diabetes, obesity and/or a prior history of preeclampsia, has demonstrated mixed results [2, 8]. A variety of sleep diagnostic tests are available, and Level I is considered the gold standard method for quantifying sleep apnoea severity but requires an overnight stay in a sleep laboratory which may not be possible for all pregnant women. Level I study set-up is completed by trained technicians, in a laboratory environment and follows the international 10–20 system (electroencephalogram (EEG), electrooculography (EOG), electromyography (EMG), snore (microphone), electrocardiogram (ECG), airflow (pressure transducer, thermistor), respiratory effort (abdomen and thoracic), oximeter (SpO_2_), sound level (dB meter), digital video (audio and visual) and position sensor)). Level II study set-up may be conducted in the patients’ home or laboratory environments and may include set-up by either trained technicians or the patient undertaking the study. Level II studies may follow either a standard or modified 10–20 system collection, similar to a Level I study, but often collects fewer EEG channels, and dependent on test location, and no digital video. Level III study set-up is typically completed by the patient in the home and have limited channel capabilities, often collecting only respiratory effort, airflow, snore, SpO_2_, though additional channels are available in some devices. Our previous publication describing these same participants in this study demonstrate a clinically interpretable agreement between Level I to self-applied Level II Somte PSG V2(Compumedics, Abbotsford, Australia) [9], and Level III Apnealink Air (Resmed, Sydney, Australia) [8] in the early to mid-gestation period (by 24 weeks). Apnealink is also validated by Facco et al. from 28 weeks’ gestation [10]. Level III tests can be completed in the home, are inexpensive, and simple to complete, however without EEG, Level III tests may provide disease severity metrics systematically different than techniques that can count arousal-based hypopneas.

To date, there are no studies exploring the pregnant participant preference of sleep diagnostic test. Engagement is important as autonomy during maternal care is associated with improved maternal and newborn health [11]. Further, there are calls for education programmes to assist healthcare professionals and childbearing women to understand women’s’ right to bodily autonomy and informed choice in maternity care [12] and for maternity services to be woman centred and responsive to consumer demand and feedback [13]. As such, the burden on pregnant women, when conducting sleep diagnostic tests should be considered, together with the preference regarding the type of where a variety of test options exist as part of a shared-decision making process.

During recruitment for a pilot randomised controlled trial which started in 2019, we observed a high rate of eligible participants declining to participate and noted a frequency of respondents citing unwillingness to complete Level I as their reason. Frequently, respondents cited family commitments as the reason they were unwilling to participate in our trial after eligibility screening. Subsequent pandemic related service shutdowns allowed us an opportunity to amend our study protocol to add self-applied unattended Level II studies, as well as a sub-study, to explore participant feedback regarding test ease of use, convenience, and repeatability, as well as participant overall preference of sleep diagnostic tests.

The aim of this study was to determine patient preferences between sleep diagnostic tests comparing Level I, Level II, Level III, in pregnant women in early to mid-gestation.

## Methods and analysis

### Study design and setting

This is a sub-study conducted during screening for eligibility of a pilot randomised controlled trial (RCT) (Australian New Zealand clinical trials registry (ANZCTR) ACTRN12619001530112), approved by South Western Sydney Local health district (SWSLHD) human research and ethics committee on June 12th, 2019 (Project identifier 2019/ETH00283), commenced in September 2019 and completed recruitment in January 2023. The current protocol version 3.1 was approved on 11th Nov 2022. All participants provided informed consent. The RCT assessed the feasibility of completing a trial assessing early intervention using continuous positive airway pressure (CPAP) or positional therapy (PT) in pregnant women with an apnoea hypopnoea index (AHI) or respiratory disturbance index (RDI) ≥ 5, *by* the 16th week of gestation. Secondary outcomes of interest in the RCT include the development of gestational diabetes mellitus (GDM) by 28 weeks gestation, the development of hypertensive disorders of pregnancy, maternal weight gain, uterine artery blood flow, glycemic control during pregnancy (in participants who develop gestational diabetes), changes in maternal circulating biomarkers and neonatal birthweight and complications.

Initially pregnant women were invited to undertake self-applied Level III tests in the home environment and attended Level I (Grael 4 K PSG: EEG, Grael Acquisition system, Compumedics, Abbotsford, Australia) at the sleep investigations unit (SIU) at Liverpool hospital (Liverpool, NSW, Australia) by the 24th week of gestation.

The addition of questionnaires and self-applied at-home Level II were approved by SWSLHD in January 2021, and implemented in March 2021. The thirty-two participants that had already been recruited at that date are not included in this analysis. Participants were screened for eligibility, invited to undertake Level I, level II, level III sleep studies and questionnaires. Participants were recruited from antenatal clinics at Campbelltown and Liverpool hospitals in South Western Sydney, NSW, Australia. Sub studies that we have analysed from this dataset assess the agreement of apnoea hypopnoea index (AHI) scores of Level I and Apnealink Air (level III) (*n* = 49) [8], and the agreement of AHI and respiratory disturbance index (RDI) of Level I and Somte (level II) (*n* = 24) [9].

### Participants

At or prior to scheduled hospital obstetric bookings, participants were approached in person or by phone by study staff who completed screening eligibility using a research electronic data capture (REDCap) hosted electronic case report form [14]. Eligible participants were provided a participant information sheet by email or hard copy and provided informed written consent. Baseline data that were collected included STOP BANG [15], Epworth Sleepiness Scale (ESS) [16] and Facco’s pregnancy-related tool questionnaire [17]. A fetomaternal physician dated gestation based on the dating scan performed in the first trimester. The study coordinator determined order of testing, based on physical availability of the test devices within a scheduled window of 7 days.

Inclusion criteria for the RCT were defined as (1) women aged 18 years of age and above; (2) In early to mid-pregnancy (up 24 weeks gestation); (3) at increased risk of metabolic complications defined as one or more of: (a) body mass index (BMI) greater than or equal to 35 kg/m^2^ at screening; (b) previous gestational diabetes mellitus (GDM) [18]; (c) previous personal history of pre-eclampsia (or in mother or sister); (d) underlying renal disease; (e) maternal type 2 diabetes (pre-gestational); (f) symptoms of sleep disordered breathing (SDB) including snoring, witnessed apnoea, mild excessive daytime sleepiness (EDS) (which does not meet the criteria for severe EDS defined by Epworth Sleepiness Scale (ESS) (> 15) or a fall asleep accident or near-miss accident in the previous 12 months) or tiredness. Participants were excluded if they have (1) previous diagnosis of OSA on active treatment; (2) confirmed current GDM or preeclampsia; (3) maternal type 1 diabetes; (4) multifetal gestation, (5) known fetal chromosomal abnormality; (6) inability to provide informed consent; (7) severe EDS based on clinical assessment (e.g. including a fall asleep motor vehicle accident or near miss, transient sleepiness while driving/at lights or needing to pull over due to sleepiness while driving, or transient sleepiness in any other dangerous situation i.e. cooking, carrying baby) or ESS of greater than 15.

Additional inclusion criteria for this sub-study were completion of any questionnaire. Participants who failed to complete any questionnaire were excluded from this analysis. Participants self-applied Level II and III sleep studies in the home environment. Assistance by family member, spouse, or other was allowed but not encouraged. The study coordinator was available on-call for technical phone support during the study set-up and data collection.

### Level I (attended PSG) (gold standard)

Participants were set up by experienced sleep technicians in the sleep investigations unit (SIU) at Liverpool Hospital. Level I data were collected between 21:00–06:00 approximately. Level I collection followed American Academy of Sleep Medicine (AASM) guidelines [19] and included EEG (C1/C2 C3/C4, O1/O2, F3/F4, M1/M2), EOG (E1/E2), EMG (chin, diaphragm and anterior tibialis (left and right)), snore (microphone), ECG (modified Lead II), airflow (pressure transducer, thermistor), respiratory effort (abdomen and thoracic), pulse oximeter (SpO2), sound level (decibel meter (dB meter)), digital video (audio and visual) and position sensor. SpO2 was collected using Compumedics adult silicone soft tip probe oximeter 3 m. EEG used gold cup electrodes (Falcon HST Snap Lead (Compumedics, Abbotsford, Australia)), EMG and ECG channels used Falcon HST Snap Leads. At times, gold cup electrodes and snap leads were used interchangeably for EEG (ground / reference), EMG (submentalis).

### Level II (unattended PSG) (Somte)

The study coordinator encouraged participants to watch a manufacturer produced YouTube instructional video prior to undertaking the test [20] and educated participants at device collection the application technique and device operation. Level II data collection followed a modified American Academy of Sleep Medicine (AASM) set-up protocol. Collection included EEG (F3/F4, M1/M2), EOG (E1/E2), EMG (submentalis, anterior tibialis (left and right leg)), ECG (modified Lead II), airflow (pressure transducer), snore, airflow (thermistor), respiratory effort (abdomen and thoracic), oximeter (SpO2) and position sensor. F3/4 leads were placed on the forehead for ease of application. SpO2 was collected using Compumedics adult silicone soft tip probe oximeter 1 m. EEG, EMG and ECG channels used Falcon HST Snap Lead.

### Level III (unattended) (Apnealink Air)

The study coordinator encouraged participants to read the manufacturer supplied instruction sheet prior to undertaking the test and educated participants at device collection the application technique and device operation. The Level III test data collection included respiratory effort, pulse, oxygen saturation (SpO2), nasal flow and snoring. Micropore tape (3 M™ Micropore™ Surgical Tape 1530–1, 3 M, North Ryde, Sydney, Australia) was issued and participants were instructed to secure air cannula and oximetry probe, using the tape, to the cheek and hand respectively to reduce the chance of lost signals.

### Questionnaires

Four questionnaires (one preferred test device questionnaire and three test device questionnaires (PSG (level I), Somte (Level II), Apnealink (Level III)) were developed in English [Online supplement 1a. PSG, 1b. Somte, 1c. Apnealink, 1d. Preferred test]. The questionnaires were beta tested prior to implementation. Beta testing was conducted on hospital staff and feedback was implemented prior to the initiation of the questionnaires. The preferred test questionnaire used a ranking scale (1st, 2nd, 3rd preferred test). Test device questionnaires used a combination of yes/no, 5-point Likert scale responses, and optional linked text fields. Level I, Level II and Level III questionnaires assessed ease of use, convenience, and repeatability on a 5-point Likert scale and used yes/no responses and linked text fields. Additionally, Level II and Level III questionnaires assessed test difficulty and phone support requirements using yes/no responses and linked test fields. Participants who required phone support during Level II and Level III sleep studies were asked to report the helpfulness of the phone support using a 5-point Likert scale.

The study coordinator scheduled questionnaires to be sent via REDcap email to participants on the morning following each scheduled test date, and following the final test, in the case of the preferred test questionnaire. Incomplete questionnaires were reissued at an interval of three days, until completion, up to a total of three times. A hard copy of each questionnaire was included with each test for all participants, and questionnaires could be completed by either hard copy or online method. Participants self-completed questionnaires, but assistance to complete questionnaires was available from the study coordinator if requested by the participant. Questionnaires completed in hard-copy form were entered into REDCap by the study coordinator.

Linked text field responses were not collected for Ease of use, convenience, and repeatability questions for Level II questionnaire due to a coding error in Redcap during questionnaire development.

### Statistical methods

Analyses were performed using Statistical Package for the Social Sciences (SPSS), Version 29.0 (IBM Corp., Chicago, Illinois, USA). Descriptive data are presented (based on distribution as assessed by a Shapiro–Wilk test) either as a mean ± standard deviation (SD), median (interquartile range (IQR) / range), or count (n (%)). Rank and Likert-scale questions were scored using Summative scale scores (sum of rank). Linked optional text fields were thematically analysed by the study coordinator and were batched according to theme. Friedman’s tests were used to calculate significance for test preference, ease of use, convenience, and repeatability. Only questionnaires completed are included in this analysis (Complete case analysis).

## Results

**Fig. 1:**
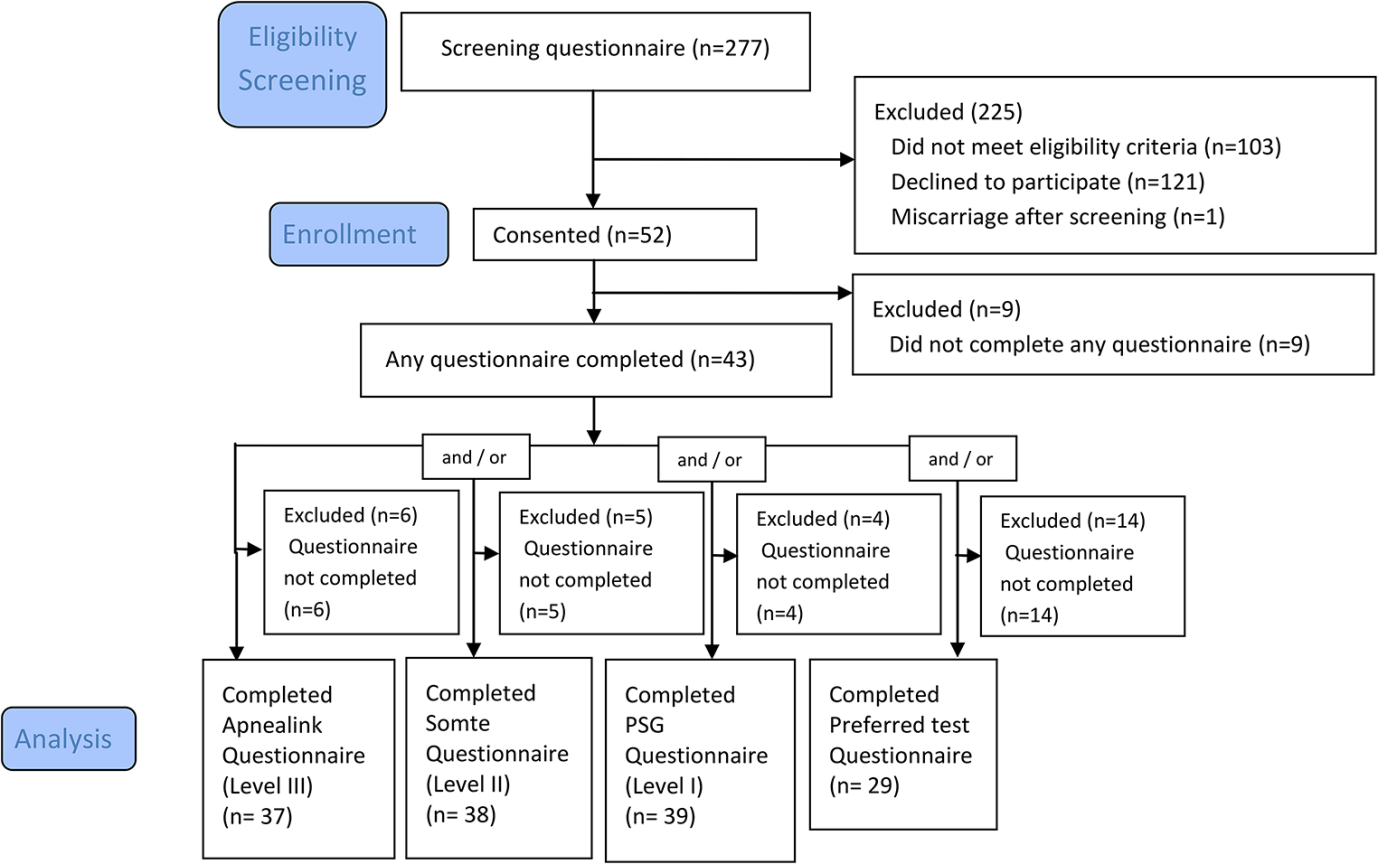
Study flow chart. Participants were excluded if they failed to complete any questionnaire. PSG represents polysomnography

A total of 277 participants were screened for eligibility after the implementation of the questionnaires and of these, fifty-two consented for participation. Nine participants were excluded from analysis due to failure to complete any questionnaire. Forty-three participants completed any questionnaire (preferred test (*n* = 29), Level III (*n* = 37), Level II (*n* = 38), and Level I (39)) and are included in this analysis (Fig. 1).

**Fig. 2:**
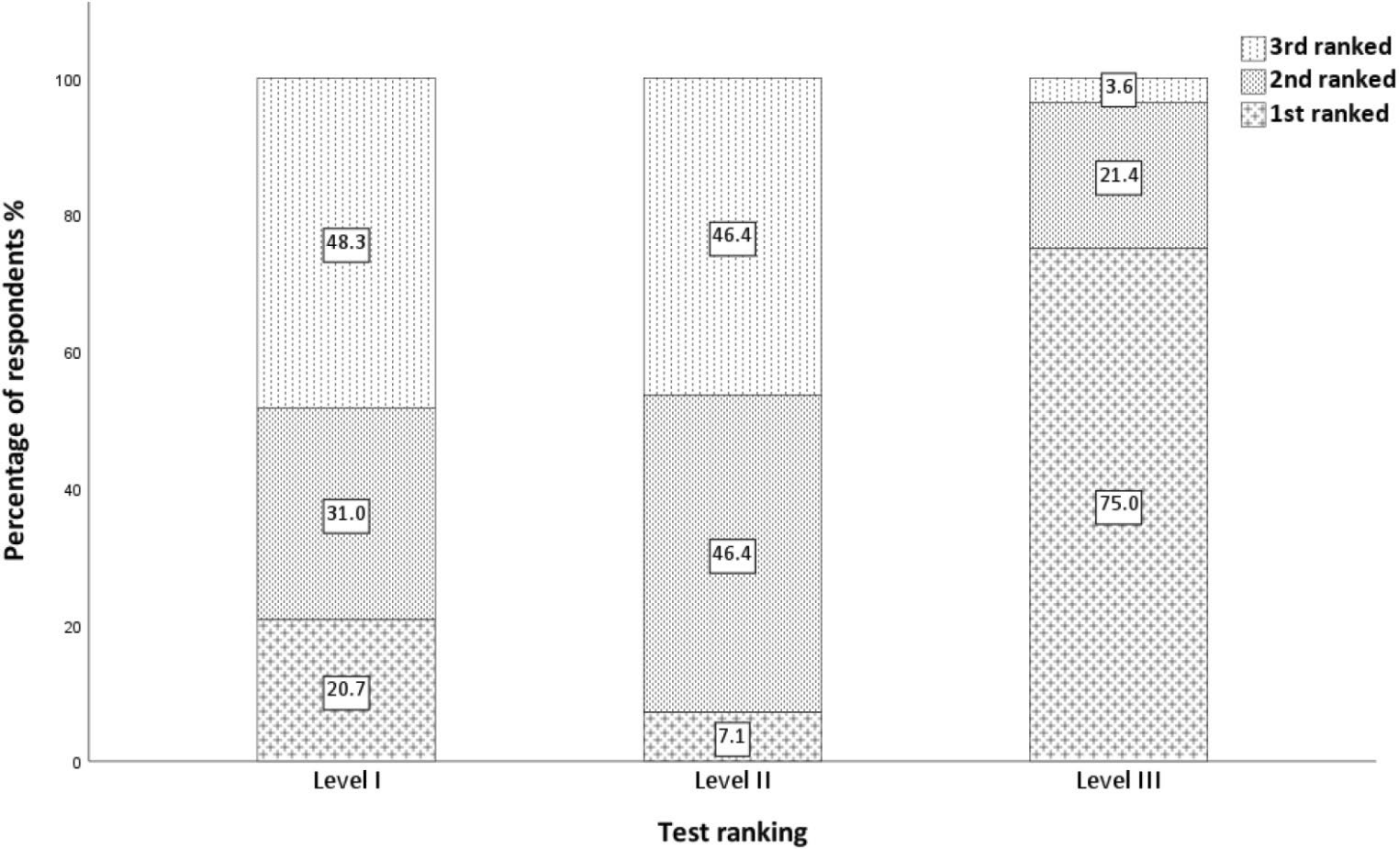
Results. Preferred test. Level I, II and III sleep studies. Level III was the preferred test (first ranked) (*n* = 21 / 28 (75.0%), compared to Level I (*n* = 6 / 29 (20.7%)) and Level II (*n* = 2 /28 (7.1%)) (*p* < 0.001))

**Fig. 3:**
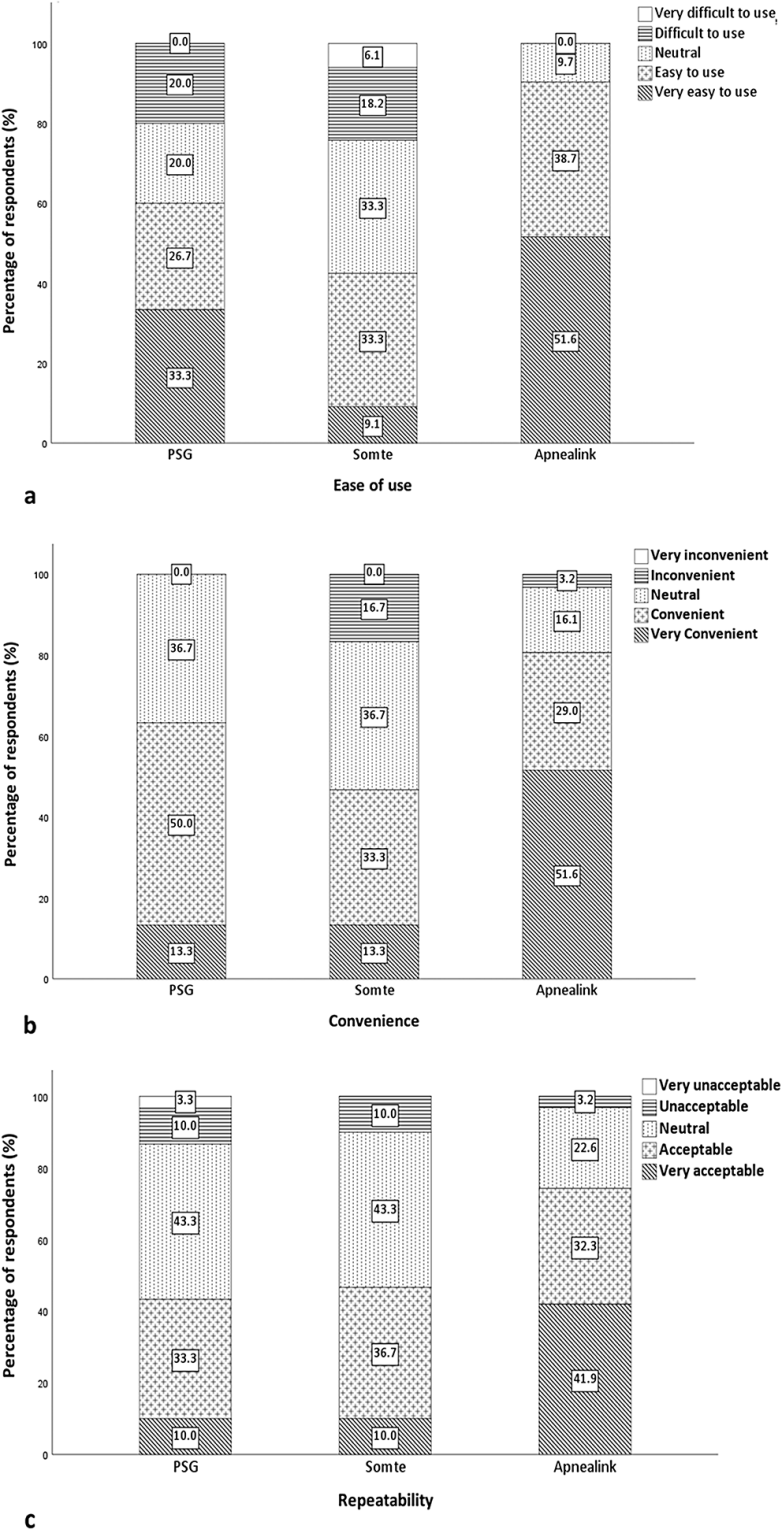
Results. **a**. Ease of use, **b**. convenience, and **c**. repeatability of Level I, Level II, and Level III. Level III was reported as the easiest to use (very easy to use) (*n* = 16 (51.6%), compared to Level I (*n* = 6 (33.3%)) and Level II (*n* = 3 (9.1%) (*p* < 0.001)) (**a**). Level III was reported as the most convenient (very convenient) (*n* = 16 (51.6%), compared to Level I (*n* = 4 (13.3%)) and Level II (*n* = 4 (13.3%) (*p* < 0.001)) (**b**). Level III was reported as the most acceptable to repeat (very acceptable) (*n* = 13 (41.9%), compared to Level I (*n* = 3 (10.0%)) and Level II (*n* = 3 (10.0%) (*p* < 0.001)) (**c**)

**Figs. 4:**
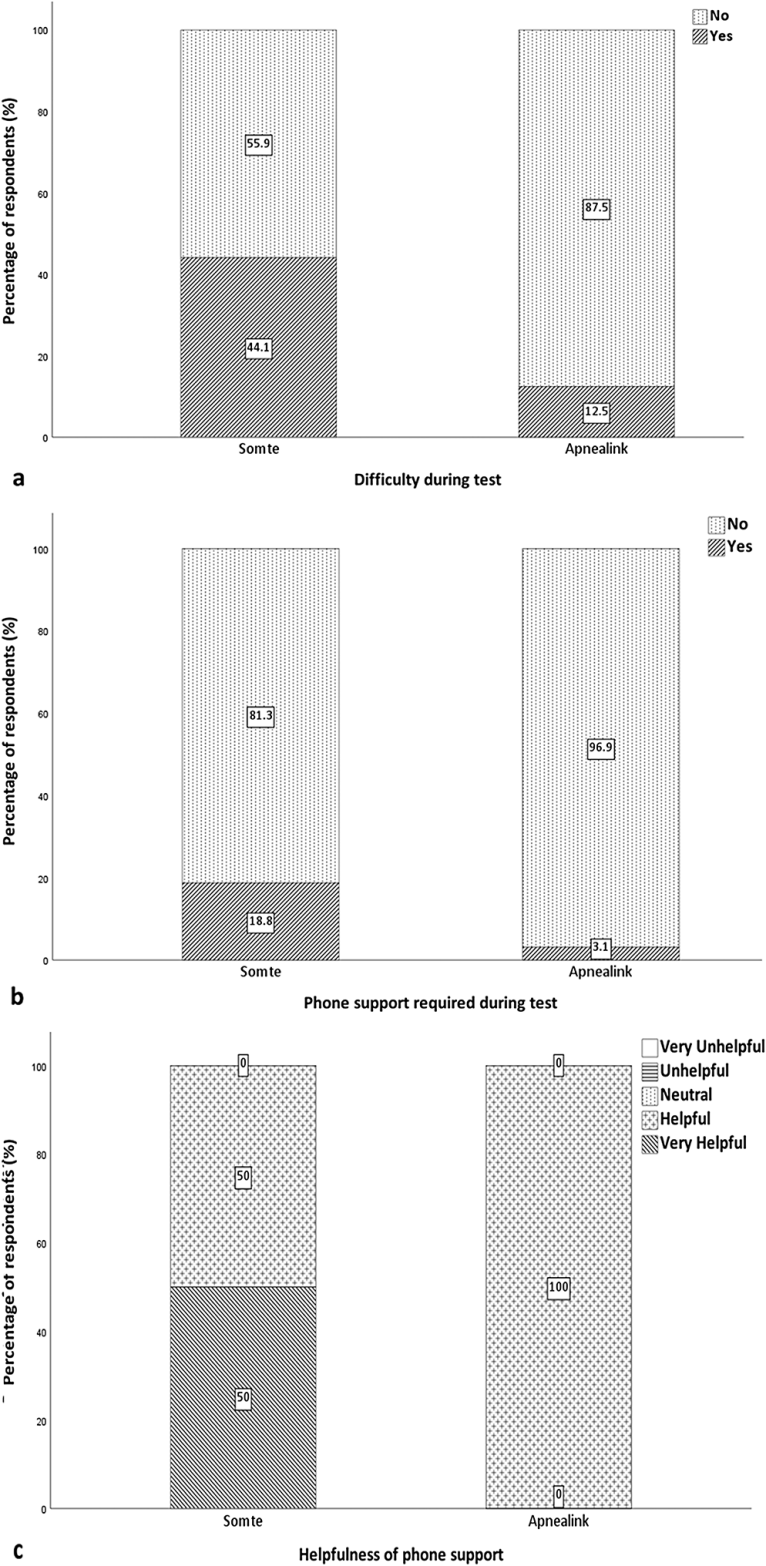
Results. **a.** Test difficulty, **b**. phone support and **c**. phone helpfulness, Level II, and Level III sleep studies. Level I was not assessed as the set-up was completed by a technician in the sleep investigations unit. Participants reported Level II as more difficult to complete (difficulty = yes) (*n* = 15 (44.1%)) than Level III (*n* = 4 (12.5%)) (**a**) and required phone support more often (phone support = yes) (*n* = 6 (18.8%)) and (*n* = 1 (3.1%)) (**b**), respectively. Participants who required phone support when completing Level II found the phone support very helpful (*n* = 3 (50%)) or helpful (*n* = 3 (50%)) and the single participant who used phone support whilst completing the Level III for the phone support helpful (*n* = 1 (100%)) (**c**)

Participant baseline demographics (*n* = 43) is shown in Table 1. Gestation at the time of the Level I test was a median of 14.2 weeks (IQR (13.5–17.6) / range (9.7–23.6)). The mean AHI for the Level I test was 3.7 (IQR (0.6–4.2) / range (0.0–20.3)). Six participants had AHI ≥ 5 on Level I (*n* = 6/30 (20%)). Preferred test questionnaire results are shown in Fig. 2. Ease of use, convenience and repeatability of test questionnaire results are shown in Fig. 3a, b, c. Linked test field written responses are shown in Online supplement 2. (Ease of use), 3. (Convenience) & 4. (Repeatability). Test difficulty and phone support results are shown in Fig. 4a, b, c, and test difficulty linked test field written responses is shown in Online supplement 6.

**Table 1:** Participant baseline demographics. (*n* = 43)

Parameter	Mean ± SD, median (IQR) or Count (%)
Age (years)	32.7 ±5.4
BMI (kg/m^2^)	30.5 ± 6.6
Nulliparity	13 (30.2%)
Parity	1 (0 - 2) / range (0 - 5)
STOP-BANG (total)	1.7 ± 1.0
ESS (total)	6.6 ± 3.8
Personal history of hypertension	15 (34.9%)
Personal history GDM	7 (16.3%)
Family history of diabetes (1st degree relative with diabetes or a sister with gestational diabetes)	24 (55.8%)
History of preeclampsia in self, mother, or sister	7 (16.3%)
Self-reported snore at least three times a week	21 (48.8%)
Witnessed apnoea’s	7 (16.3%)
Ethnicity	Caucasian 16 (37.2%) Other 7 (16.3%)
	Asian 5 (11.6%)
	Indian/Subcontinental 4 (9.3%)
	Middle Eastern 4 (9.3%)
	Aboriginal/Torres 4 (9.3%) Strait Islander Polynesian 3 (7.0%)

BMI represents body mass index. ESS represents Epworth sleepiness scale. GDM represents gestational diabetes mellitus

Of the participants who did not complete the Level I test, the reason provided for non-completion of the test included commitments (other) (*n* = 1), family commitments (*n* = 3), Covid (*n* = 3), travel (*n* = 1) and no reason provided (*n* = 5) [Online supplement 5a]. Of the participants who did not complete the level II test, the reason provided for non-completion of the test included commitments (other) (*n* = 1), Illness (*n* = 1), family commitments (*n* = 1), difficulty completing test and tiredness (*n* = 1) and no reason provided (*n* = 10) [Online supplement 5b]. Of the participants who did not complete Level III test, the reason provided for non-completion of the test included commitments (other) (*n* = 1), family commitments (*n* = 1), illness (*n* = 1), tiredness (*n* = 1) and no reason provided (*n* = 6) [Online supplement 5c].

## Discussion

Pregnant women in early to mid-gestation preferred to complete Level III sleep studies compared to Level II and Level I. Level III was rated easier to use, more convenient and more acceptable to repeat. Most participants in this study preferred the Level III test, which we had anticipated, however, surprisingly there were a number who preferred the Level I and II tests.

More than half of the respondents rated the Level III test as very easy to use, and this reflects the simplicity of the test, with its simple-to-follow set up process which was cited by all respondents. More than half of the respondents ranked the Level III test very convenient, and respondents cited set up process and location as the reason for the response. The Level I test scored higher than Level II on all domains apart from repeatability. Technician set up during the Level I test was cited most frequently as the reason for the high score for ease of use and convenience. Prior to undertaking this study, we had assumed the Level II test in the home environment would be favoured over the laboratory-based Level I test, however, this was not the case, and several participants verbally reported enjoying sleeping away from the home environment in the hospital sleep unit. The lower score across most domains for the Level II test reflects the fact that pregnant women found this difficult to complete at home without technician set-up. Nearly half of the participants who completed the Level II test reported difficulty completing the test, with the highest cited reason the complicated test set-up. Whilst we did not encourage assistance by spouse or other whilst completing the Level II set-up process in the home, some participants in this study sought help from a spouse or other person during the set-up process, though it is unclear to what extent this affected the participant responses.

The Level III test was rated very acceptable to repeat by nearly half of the respondents. Compared to just 10% of respondents who reported the Level I or Level II test very acceptable to repeat, the high rating for repeatability of the Level III test likely reflects the reduced burden of the Level III test compared to Level I and Level II tests. This is important, as OSA severity increases with advancing gestation [5, 21], which may necessitate repeat testing during the pregnancy in some women. Additionally, given postpartum testing is recommended in women found to have OSA during pregnancy [2], The Level III test provides an option for repeat testing during the pregnancy or in the post-partum period, that is easy, convenient, and acceptable to undertake. Whilst technician set-up during the Level I test increased ease of use and convenience of the test compared to the Level II test, this did not translate to high repeatability of the Level I test, with only 10% of respondents citing the Level I test as very acceptable to repeat, with simply not wanting to retake the test as the highest reason for the score.

The main limitation of this study was the small sample size, which was not prespecified as our intention was to collect feedback from as many participants as possible whilst the study was operational. This study was interrupted several times in 2021–2022 due to pandemic-related public health orders and related study suspensions, and this severely impacted our capacity to recruit participants for our study. Further, nearly half of eligible participants declined consent, and though we did not collect data on the reasons for this, the leading verbal reason for unwillingness to participate was the requirement for the Level I test. Of the consented participants who did not complete the tests, family commitments SARS CoV-2 were most frequently cited as the reason for non-completion, which reflects both the ongoing nature of responsibilities faced by families, and the momentary burden SARS CoV-2 placed on clinical and research services, highlighting the importance of participant engagement when designing research studies in this and other similar population. This study did not collect demographic data on socioeconomics, income, education, or relationship status of the participants in this study. We acknowledge these factors could influence the responses of the participants and therefore affect the generalisability of these results.

A further limitation in this study was the technical failure of the linked field open ended response options in the Level II test questionnaires for ease of use, repeatability, and convenience. This was not detected until after questionnaire implementation and as such, we are unable to provide reasons for participant ratings in these domains. Whilst the optional written responses were not captured, the Likert scale responses for all questions, plus the linked field text responses for the Level II study for test difficulty were, thus this technical failure does not present an overt failure of the study.

The participants in this study preferred Level III tests, and this is not surprising given the simplicity of the test and home location for testing. There are, however, instances where Level I is recommended, such as in those with suspected movement disorders of sleep, neuropsychological disorders, disabilities that prevent unattended sleep studies, significant cardiorespiratory disease and those requiring additional monitoring i.e. video and/or carbon dioxide (CO_2_) monitoring [19, 22]. Recent guidelines suggest unattended sleep studies may be appropriate for testing in pregnancy [2], and both Level II and III studies have been validated in the pregnant population in both early-mid and later gestation. However, level II and III tests are not adequate in all instances. Our group has previously reported a diagnosis of rapid eye movement (REM) sleep without atonia (RSWA) during pregnancy, in a woman with no reported history of movement disorders of sleep [9], and the significance of this incidental finding is still yet unclear but does highlight the potential for this occurrence during pregnancy. Additionally, we have reported AHI in early gestation as high as 119.7 events per hour, demonstrating a potential requirement for CO_2_ monitoring in pregnant women who may be suspected of hypoventilation. As such, despite the preference of Level III tests in this small sample, we caution clinicians against only offering this test.

Participants in this study completed Level I, II and III studies, providing a rare insight into the participant experience across three sleep study types. The sleep diagnostic tests in this study were completed in early to mid-gestation, providing a window of opportunity for early detection of OSA in pregnancy, and allowing early implementation of therapy and/or safe delivery planning. The screening of at-risk pregnancies is recommended in the early to mid-gestation period (6–28 weeks) [2] and our study includes participants who were all within this gestational range, making the findings of this study particularly invaluable for clinical and research services investigating OSA in pregnancy.

Finally, though this study demonstrates pregnant women prefer to undertake Level III sleep studies compared to Level II or Level I, currently in Australia there is no reimbursement scheme for Level III sleep diagnostic tests. We therefore acknowledge the difficulty in translating the findings of this study into clinical practice in our region.

## Conclusion

Level III sleep studies were preferred by pregnant women in early to-mid gestation, though a small proportion preferred Level I or II studies. Future clinical and research services investigating OSA in pregnancy could consider the appropriateness of each test in each case, and where clinically appropriate, involve pregnant women in shared decision making regarding test type, thus providing an opportunity to improve maternal care in women undertaking sleep diagnostic studies in the future.

## Electronic supplementary material

Below is the link to the electronic supplementary material.

Supplementary Material 1

Supplementary Material 2

Supplementary Material 3

Supplementary Material 4

Supplementary Material 5

Supplementary Material 6

## Data Availability

Data will be made available on reasonable request to the first or last authors.
