# Supplementary material for: Patient preference of level I, II and III sleep diagnostic tests to diagnose obstructive sleep apnoea among pregnant women in early to mid-gestation
Source: Sleep Breath. 2024 Aug 21;28(6):2387–95. doi: 10.1007/s11325-024-03114-0 (PMC11568020; doi:10.1007/s11325-024-03114-0)
Supplement: Supplementary file 1 — Supplementary Material 1 [file 11325_2024_3114_MOESM1_ESM.pdf]

/

Laboratory based sleep study up to 24 weeks gestation

Please complete the survey below regarding the hospital sleep laboratory study

Thank you!

Did you complete the Hospital Laboratory Sleep Study?	◯ Yes<br>◯ No
If No, please provide reason (optional_	__________________________________
Overall, how was the Hospital Laboratory Sleep Study? (Ease of use)	◯ Very difficult to use<br>◯ Difficult to use<br>◯ Neutral<br>◯ Easy to use<br>◯ Very easy to use
Why? (optional)	__________________________________
Overall, how was the Hospital Laboratory Sleep Study? (Convenience)	◯ Very inconvenient<br>◯ Inconvenient<br>◯ Neutral<br>◯ Convenient<br>◯ Very Convenient
Comments? (optional)	__________________________________
If required, how acceptable would you find repeating this test?	◯ Very unacceptable<br>◯ Unacceptable<br>◯ Neutral<br>◯ Acceptable<br>◯ Very acceptable
What were the main reasons for your answer? (optional)	__________________________________

Thankyou for completing this questionnaire!

Somte sleep study up to 24 weeks gestation-questionnaire

Please complete the survey below relating to the SOMTE home sleep study

Thank you!

Did you complete the Somte home test?	◯ Yes<br>◯ No
If No, please provide reason (optional_	__________________________________
Did you have difficulty completing the SOMTE Home Sleep Study	◯ Yes<br>◯ No
If Yes, please describe the difficulty (optional)	__________________________________
Did you need any phone support during the SOMTE Home Sleep Study?	◯ Yes<br>◯ No
If you used phone support, was the phone support helpful?	◯ Very Unhelpful<br>◯ Unhelpful<br>◯ Neutral<br>◯ Helpful<br>◯ Very Helpful
Comments? (Optional)	__________________________________
Overall, how was the SOMTE home sleep study? (Ease of use)	◯ Very difficult to use<br>◯ Difficult to use<br>◯ Neutral<br>◯ Easy to use<br>◯ Very easy to use
Why? (optional)	__________________________________

projectredcap.org

Overall, how was the SOMTE home Sleep Study? (Convenience)	◯ Very inconvenient<br>◯ Inconvenient<br>◯ Neutral<br>◯ Convenient<br>◯ Very Convenient
Comments? (optional)	__________________________________
If required, how acceptable would you find repeating this test?	◯ Very unacceptable<br>◯ Unacceptable<br>◯ Neutral<br>◯ Acceptable<br>◯ Very acceptable
What were the main reasons for your answer? (optional)	__________________________________

Thankyou for completing this questionnaire!

Apnealink up to 24 weeks gestation questionnaire

Please complete the survey below regarding the home apnealink sleep study

Thank you!

Did you complete the apnealink home test?	◯ Yes<br>◯ No
If No, please provide reason (optional)	____
Did you have difficulty completing the apnealink Home Sleep test?	◯ Yes<br>◯ No
If Yes, please describe the difficulty (optional)	____
Did you need any phone support during the apnealink Home Sleep test?	◯ Yes<br>◯ No
If you used phone support, was the phone support helpful?	◯ Very Unhelpful<br>◯ Unhelpful<br>◯ Neutral<br>◯ Helpful<br>◯ Very Helpful
Comments? (Optional)	____
Overall, how was the Apnealink home sleep test? (Ease of use)	◯ Very difficult to use<br>◯ Difficult to use<br>◯ Neutral<br>◯ Easy to use<br>◯ Very easy to use
Why? (optional)	____
Overall, how was the Apnealink home Sleep test? (Convenience)	◯ Very inconvenient<br>◯ Inconvenient<br>◯ Neutral<br>◯ Convenient<br>◯ Very Convenient
Comments? (optional)	____

projectredcap.org REDCap

If required, how acceptable would you find repeating this test?	◯ Very unacceptable<br>◯ Unacceptable<br>◯ Neutral<br>◯ Acceptable<br>◯ Very acceptable
What were the main reasons for your answer? (optional)	__________________________________

Thankyou for completing this questionnaire!

projectredcap.org REDCap

Preferred test questionnaire

Please complete the survey below

Thank you!

Please rank the three tests in order of preference 1=most preferred, 3= least preferred

		1	2	3
1)	Hospital laboratory sleep study	○	○	○
2)	Home sleep study SOMTE	○	○	○
3)	Home sleep study Apnealink	○	○	○
