# Supplementary material for: Patient preference of level I, II and III sleep diagnostic tests to diagnose obstructive sleep apnoea among pregnant women in early to mid-gestation
Source: Sleep Breath. 2024 Aug 21;28(6):2387–95. doi: 10.1007/s11325-024-03114-0 (PMC11568020; doi:10.1007/s11325-024-03114-0)
Supplement: Supplementary file 2 — Supplementary Material 2 [file 11325_2024_3114_MOESM2_ESM.pdf]

Response	Theme
Because the lady helped put it on	Set up (Technician assistance)
bed not comfortable	Comfort
Easy in terms of just having to sit there while being wired up. Not easy in terms of functionality and being able to sleep.	Technician assistance
Found it very hard to be comfortable and fall asleep	Comfort
I didn't have to put any of the equipment on or monitor that it was working (myself)	Set up (Technician assistance)
I had a good sleep doesn't my sleeping at night.	Other
it was ok, just a lot of wires	Comfort
it wasn't too easy to sleep with those wires	Comfort
it wasn't too uncomfortable, but it was itchy and I got tangled a few times during the night	Comfort
not difficult but uncomfortable but helpful	Comfort
Staff set everything up for me.	Set up (Technician assistance)
The staff where great and explained how things were going to go	Set up (Technician assistance)
The wires fell off, the finger sensor stopped working, took a long time to set up	Other
very easy as technician set up device	Set up (Technician assistance)

Online supplement 2a.

Response	Theme

Online supplement 2b.

Response	Theme
Instructions were clear, not many different parts	Set up (self-application)
instructions were clear/ easy to follow	Set up (self-application)
It was easy to follow and put on but it was just too uncomfortable especially when it's attached to your chest and waist and cable stabbing into the side of your body	Set up (self-application) / comfort
My husband was helping putting on the machine while Im relax and ready to start.	Set up (self-application)
Not much to do in regard to setting up.	Set up (self-application)

Online supplement 2c.

Online supplement 2. Participant responses to linked field (Ease of use). 2a. PSG (polysomnography), 2b. Somte, and 2c. PSG. Linked field responses and themes. Participant responses for Somte were not captured due to a coding error in the questionnaire.
