# Supplementary material for: Patient preference of level I, II and III sleep diagnostic tests to diagnose obstructive sleep apnoea among pregnant women in early to mid-gestation
Source: Sleep Breath. 2024 Aug 21;28(6):2387–95. doi: 10.1007/s11325-024-03114-0 (PMC11568020; doi:10.1007/s11325-024-03114-0)
Supplement: Supplementary file 3 — Supplementary Material 3 [file 11325_2024_3114_MOESM3_ESM.pdf]

Response	Theme
Depends on definition of convenience. In terms of process and organisation was highly convenient. In terms of physically being able to sleep this was not convenient.	Set up (Technician assistance) / Comfort
Easy with work and staff were prompt to answer buzzer when I needed to get up during the night.	Set up (Technician assistance)
everyone is very helpful, clean room. its just the wires	Set up (Technician assistance) / Comfort
Had to leave home for the test, but once there was set up by staff	Set up (Technician assistance) / Location
I could come after work and choose which day that also suited me	Other
Just the rush to get out didn't enjoy that although the 2 lady's that helped me out were lovely	Set up (Technician assistance) / Other
Nurses were very helpful to comfort the patient before doing the sleep study machine.	Set up (Technician assistance)
work at hospital, don't want to sleep here	Location

Online supplement 3a.

Response	Theme

Online supplement 3b.

Response	Theme
Didn't take time to set up	Set up (self-application)
Easy to put on and monitor my sleeping at night.	Set up (self-application)
I wouldn't want to do it for over a 24hr period	Other
It allowed me to complete the sleep study at home which was very convenient.	Location
with the steps in the booklet it was very convenient	Set up (self-application)

Online supplement 3c.

Online supplement 3. Participant responses to linked field (Convenience). 3a. PSG (polysomnography), 3b. Somte, and 3c. Apnealink. Linked field responses and themes. Participant responses for Somte were not captured due to a coding error in the questionnaire.
