# Supplementary material for: Patient preference of level I, II and III sleep diagnostic tests to diagnose obstructive sleep apnoea among pregnant women in early to mid-gestation
Source: Sleep Breath. 2024 Aug 21;28(6):2387–95. doi: 10.1007/s11325-024-03114-0 (PMC11568020; doi:10.1007/s11325-024-03114-0)
Supplement: Supplementary file 4 — Supplementary Material 4 [file 11325_2024_3114_MOESM4_ESM.pdf]

Responses	Theme
as I don't have to take time off work, and it was easy to do in hospital	Set up (Technician assistance) / Location
Being away from family, disruption to routine and sleep	Location / Disruptive
dont want to do it	Do not want to repeat
i can sleep the same hours as normal	Convenient
I don't want to repeat, but not the worst thing in the world to redo	Do not want to repeat
I felt I didn't sleep.	Poor sleep
I need support to my health especially when I sleep.	Beneficial to sleep
I need to allocate a good time for when my husband is home as he works shift work.	Location /
id not love to, but I can if really required	Do not want to repeat
It was an uncomfortable situation and almost impossible to sleep with the level of equipment.	Comfort / Poor sleep
it was hard with the headache i have this morning	Comfort
not sure as a bit uncomfortable	Comfort
Test was fine but I would prefer to not do this test again as it isn't that comfortable.	Do not want to repeat / Comfort
as I don't have to take time off work, and it was easy to do in hospital	Set up (Technician assistance) / Location

Online supplement 4a.

Responses	Theme

Online supplement 4b.

Responses	Theme
Disruption to routine and sleep	Poor sleep / Disruptive
Get support for my health	Beneficial to health
good to do at home	Location
I would do it if it beneficial to me and my baby.	Beneficial to health
it didnt take very long at all to set up and go through the instructions making it a lot more convenient to use	Set up (self-application)
nasal prongs uncomfortable	Comfort
This was the easiest to set up and most comfortable	Comfort / Set up (self-application)
Wasn't too bad The most uncomfortable part was the finger sensor, which was irritating during the night ad would slip and needed adjusting	Disruptive

Online supplement 4c.

Online supplement 4. Participant responses to linked field (Repeatability). 4a. PSG (polysomnography), 4b. Somte, and 4c. Apnealink. Linked field responses and themes. Participant responses for Somte were not captured due to a coding error in the questionnaire.
