# Supplementary material for: Patient preference of level I, II and III sleep diagnostic tests to diagnose obstructive sleep apnoea among pregnant women in early to mid-gestation
Source: Sleep Breath. 2024 Aug 21;28(6):2387–95. doi: 10.1007/s11325-024-03114-0 (PMC11568020; doi:10.1007/s11325-024-03114-0)
Supplement: Supplementary file 5 — Supplementary Material 5 [file 11325_2024_3114_MOESM5_ESM.pdf]

Response	Theme
children at home (family committment)	Family commitments
COVID	Covid
Covid sleep lab closure	Covid
family committments	Family commitments
family commitments	Family commitments
not a goodtime	Commitments
positive covid test at sleep lab	Covid / Illness
too far to travel	Travel

Online supplement 5a.

Response	Theme
family committments	Family commitments
it was too complicated and overwhhelming	Difficulty completing
not a good time	Tiredness
too complicated/ zero sleep	Difficulty completing / Tiredness
unwell on the night	Illness

Online supplement 5b.

Response	Theme
family committments	Family commitments
not a good time	Commitments
too tired	Tiredness
unwell	Illness

Online supplement 5c.

Online supplement 5. Participant responses for non-completion of test. 5a. PSG, 5b. Somte and 5c. Apnealink. Linked field responses and themes.
