# Supplementary material for: Patient preference of level I, II and III sleep diagnostic tests to diagnose obstructive sleep apnoea among pregnant women in early to mid-gestation
Source: Sleep Breath. 2024 Aug 21;28(6):2387–95. doi: 10.1007/s11325-024-03114-0 (PMC11568020; doi:10.1007/s11325-024-03114-0)
Supplement: Supplementary file 6 — Supplementary Material 6 [file 11325_2024_3114_MOESM6_ESM.pdf]

Responses	Theme
battery ran out before I started and I had to chage it and it had died when I woke up	Equipment issue
cat jumped on bed while doing test	Other
could not sleep. removed test early	Discomfort
did not turn on	Equipment issue
Didn't have enough of the sticky press studs to complete the test as scheduled. Had to have the machine reset to complete	Equipment issue
Getting everything together and putting wires in	Complicated
Had to remove it at 2:30am as it was so uncomfortable.	Discomfort
it was too much to do	Complicated
it was uncomfortable	Discomfort
quite complicated, got assistance from my husband	Complicated
the machine didn work when i tried to turn it on	Equipment issue
Took longer than expected to connect all the wires. Some instructions were slightly different to what was provided.	Complicated
Uncomfortable	Discomfort
Was a bit confused with the wires to go on the legs of where they should sit	Complicated

Online Supplement 6a.

**Responses**	**Theme**
device came off first night. oximtere was tight	Equipment issue
It was uncomfortable	Discomfort
understanding the cables	Complicated

Online Supplement 6b.

Online Supplement 6. Participant responses to linked field (Difficulty). 6a. Somte, and 6b. Apnealink. Linked field responses and themes.
